# THE GSA PRESIDENTIAL SYMPOSIUM — STRENGTH IN AGE: HARNESSING THE POWER OF NETWORKS

**DOI:** 10.1093/geroni/igz038.2362

**Published:** 2019-11-08

**Authors:** Harvey J Cohen

**Affiliations:** Duke University Medical Center, Durham, North Carolina, United States

## Abstract

GSA is a network of disciplinary components each of which is a network composed of members. This large system possesses powerful emergent properties that are realized at many interdisciplinary interfaces. The GSA Presidential Symposium mirrors this by bringing together rapporteurs from our disciplinary presidential symposia who will summarize the major findings of these symposia. The Behavioral and Social Sciences presidential symposium explores the use of social networking by aging adults, the media and technologies utilized, and the impact of these trends on health and well-being. The Biological Sciences presidential symposium examines multiple layers of biological networks as predictors of systemic aging. The Social Research Policy and Practice presidential symposium dissects the reciprocal relationship between broad environmental contexts and social networks throughout life and ways in which this relationship can be used to optimize the aging experience. The Health Sciences presidential symposium reports on the improvement of perioperative care of older adults achieved through harnessing the partnership and collaboration of all disciplines involved in this care. The Academy of Gerontology in Higher Education presidential symposium discusses ongoing initiatives that build networks to shape age-friendly programs and policies at universities, international collaborations, and competency-based gerontology education. The Humanities and Arts presidential symposium investigates the synergy between museums and aging, both of which are represented as networks. A discussion between the presidential symposia rapporteurs with audience participation will ensue to identify common, overarching themes and to spark approaches to important, interdisciplinary problems in gerontology. • Kelly Niles-Yokum, Academy for Gerontology in Higher Education, Education Networks: Strengthening of Gerontology and Geriatrics through Connectivity • Kristine J. Ajrouch, Behavioral and Social Sciences Section, The Ties That Bind: The Influence of Social Media and Technology in the Lives of Older Adults • George L. Sutphin, Biological Sciences Section, Expanding the Geroscience Network • Luigi Ferrucci, Health Sciences Section, Optimizing Surgical Care for All Older Adults • Kate de Medeiros, Humanities and Arts Committee, Museums and Aging: Novel Network Opportunities to Support Optimal Aging • Emily Greenfield, Social Research Policy and Practice Section, Harnessing Social Networks to Optimize Environmental Contexts for Diverse Aging Experiences

